# An innovative virtual fellowship leveraging global and regional mentorship to foster pediatric neuro-oncologists in low/middle-income countries

**DOI:** 10.1093/noajnl/vdaf229

**Published:** 2025-10-23

**Authors:** Zeena Salman, Daniel C Moreira, Rahat Ul Ain, Julieta Hoveyan, Alma Edith Benito Resendiz, Ludi Dhyani Rahmartani, Anan Zhang, Nisreen Amayiri, Simon Bailey, Eric Bouffet, Godfrey Chi-Fung Chan, Anthony Pak-Yin Liu, Andres Morales La Madrid, Naureen Mushtaq, Karen Tsui, Thandeka Vuyiswa Zamansundu Ngcana, Mauricio Sanchez Salazar, Vasudeva Bhat K, Ramona Cirt, Mahendra Somathilaka, Peiyi Yang, Girish Chinnaswamy, Girish Dhall, Tejpal Gupta, Rakesh Jalali, Alvaro Lassaletta, Diana S Osorio, Margaret Shatara, Santhosh A Upadhyaya, Ramya Uppuluri, Stefan Pfister, Susan Ybarra, Elizabeth DiNovis, Carlos Rodriguez-Galindo, Ibrahim Qaddoumi

**Affiliations:** Department of Global Pediatric Medicine, St Jude Children’s Research Hospital, Memphis; Department of Global Pediatric Medicine, St Jude Children’s Research Hospital, Memphis; Department of Oncology, St Jude Children’s Research Hospital, Memphis; Department of Pediatric Hematology/­Oncology, University of Child Health Sciences, The Children’s Hospital Lahore, Lahore; Department of Pediatric Oncology, Pediatric Cancer and Blood Disorders Center of Armenia, Yeolyan Hematology and Oncology Center, Yerevan; Department of Pediatric Research, Immune Oncology Research Institute, Yerevan (J.H.); Department of Pediatric Oncology, National Medical Center “20 de Noviembre,” ISSSTE, Mexico City; Pediatric Hematology Oncology Division, Department of Child Health, Faculty of Medicine, Universitas Indonesia—Dr Cipto Mangunkusumo Hospital, Jakarta; Hematology and Oncology Department, Shanghai Children’s Medical Center, Shanghai Jiao Tong University School of Medicine, Shanghai; Division of Pediatric Hematology/Oncology, King Hussein Cancer Center, Amman; Department of Paediatric Oncology, Sir James Spence Institute of Child Health, Newcastle upon Tyne; Wolfson Childhood Cancer Research Centre, Newcastle University Centre for Cancer, Newcastle upon Tyne; Department of Paediatrics, University of Toronto, Toronto; Department of Paediatrics and Adolescent Medicine, School of Clinical Medicine, The University of Hong Kong, Hong Kong Special Administrative Region; Department of Paediatrics, University of Toronto, Toronto; Neuro-Oncology Unit, Pediatric Cancer Center Barcelona, Sant Joan de Deu Children’s Hospital, Barcelona; Department of Oncology, Aga Khan University Hospital, Karachi; Department of Paediatric Haematology/Oncology, Starship Children’s Hospital, Auckland; Paediatric Haematology-Oncology Unit, Chris Hani Baragwanath Academic Hospital, Wits Donald Gordon Medical Centre, University of the Witwatersrand, Johannesburg; Pediatric Oncology Department, Hospital Nacional de Niños, San José; Department of Pediatric Oncology, Kasturba Medical College, Manipal, Manipal Academy of Higher Education; Department of Pediatric Oncology, “Marie S. Curie” Children’s Hospital, Bucharest; Pediatric Oncology Unit, National Cancer Institute, Maharagama; Medical Oncology Department, Pediatric Oncology Center, Beijing Children’s Hospital, Capital Medical University, National Center for Children’s Health, Beijing; Department of Medical Oncology, Tata Memorial Centre, Mumbai and Homi Bhabha National Institute, Mumbai; Division of Pediatric Hematology, Oncology, and Blood & Marrow Transplantation, University of Alabama at Birmingham (UAB), Birmingham; Department of Radiation Oncology, ACTREC, Tata Memorial Centre, HBNI, Navi Mumbai; Department of Radiation Oncology, Apollo Proton Cancer Center, Chennai; Pediatric Neuro-Oncology Unit, Hospital Infantil Universitario Niño Jesús, Madrid; Department of Pediatrics, The University of Texas MD Anderson Cancer Center, Houston; Department of Pediatric Hematology and Oncology, Children’s Hospitals and Clinics of Minnesota, Minneapolis; Department of Pediatrics and Communicable Diseases, Division of Pediatric Hematology Oncology, C. S. Mott Children’s Hospital, University of Michigan Medical School, Ann Arbor; Department of Pediatric Hematology, Oncology, BMT, Apollo Hospitals, Chennai; Division of Pediatric Neurooncology, German Cancer Research Center (DKFZ), German Cancer Consortium (DKTK), and KiTZ Clinical Trial Unit, Heidelberg University Hospital, Heidelberg; Department of Global Pediatric Medicine, St Jude Children’s Research Hospital, Memphis; Department of Global Pediatric Medicine, St Jude Children’s Research Hospital, Memphis; Department of Global Pediatric Medicine, St Jude Children’s Research Hospital, Memphis; Department of Oncology, St Jude Children’s Research Hospital, Memphis; Department of Global Pediatric Medicine, St Jude Children’s Research Hospital, Memphis; Department of Oncology, St Jude Children’s Research Hospital, Memphis

**Keywords:** capacity building, CNS tumors, low- and middle-income countries, neuro-oncology, pediatric, virtual fellowship

## Abstract

**Abstract:**

BackgroundMost children with central nervous system (CNS) tumors reside in low- and middle-income countries (LMICs), with limited availability of trained pediatric neuro-oncologists.

**Methods:**

Using a series of structured interviews of physicians who had served as global mentors or mentees in pediatric oncology, we identified mentorship, leadership, and clinical training as key components necessary to virtually train pediatric oncologists in LMICs to become leading pediatric neuro-oncologists while they remain in their home countries. Thus, the St Jude Global Virtual Pediatric Neuro-oncology Fellowship (VPNOF) was designed to incorporate mentorship with global and loco-regional mentors to aid in each fellow’s career and institutional goal setting and clinical training involving virtual tumor boards and didactics and ad-hoc case discussions, enabling fellows to manage patients at their home institution. Fellows traveled to their mentors’ institutions twice for four-week clinical rotations.

**Results:**

In 2022 and 2023, eleven fellows were selected, representing 10 LMICs. The 2-year fellowship led to the establishment of multi-disciplinary approaches, increased patient volume, increased use of evidence-based practices, 33 abstract presentations, and publication of four journal articles.

**Conclusions:**

The VPNOF is an innovative approach leveraging global mentorship to train pediatric oncologists in resource-limited settings to become pediatric neuro-oncologists, which has led to the successful implementation of new practice paradigms to improve the quality of care for children with CNS tumors in LMICs.

Key Points

- The St Jude Global Virtual Pediatric Neuro-oncology Fellowship was developed to train pediatric oncologists in low- and middle-income countries.
- The fellowship led to improved patient volume and increased use of evidence-based practices.

Pediatric brain tumors are the second most common malig­nancy in children and adolescents, and the leading cause of cancer-related childhood mortality.^1^ The fact that 80%-90% of the world’s children reside in low- and middle-income countries (LMICs) contributes to a large survival disparity, with rates of <40% in some LMICs versus 70%-80% in higher-income coun­tries.^2^ In fact, 1 of the 6 index cancers identified by the WHO in its Global Initiative Childhood Cancers is low-grade glioma.^3^ Therefore, addressing disparities in neuro-oncological care is critical to improving overall pediatric cancer survival globally.

The successful management of children with CNS tumors warrants the incorporation of a multidisciplinary team in the care of the child. However, countries with limited resources often lack access to the training and resources required to develop pediatric neuro-oncologists and multidisciplinary teams.^4^ The absence of such specialized, multidisciplinary care is a cause of the gap in survival and neurologic functional outcomes of children with CNS tumors in LMICs and those in high-income countries.^5^^,^^6^

St Jude Global has multiple training programs, and this fellowship takes a different approach via virtual training.^7^ Virtual consultations and education have emerged—particularly since the COVID era—as effective and convenient approaches to medical consultation and distance learning,^8^ making them promising methods for providing cost-effective education to healthcare professionals in LMICs. A virtual fellowship in a highly specialized field such as pediatric neuro-oncology (PNO) is a novel approach to bridging the gap in the training and mentorship necessary to manage CNS tumors in children across the globe. Such virtual training initiatives enable LMICs to keep their experts during the training and increase chances of retaining them after training and build local capacities compared to their likelihood of returning to their home country after in-person training in higher-income countries.^9^

Importance of the Study

Most children with central nervous system (CNS) tumors live in low- and middle-income countries (LMICs), where there is a shortage of trained pediatric neuro-oncologists. Structured interviews with physicians highlighted mentorship, leadership, and clinical training as being essential for developing local expertise. We created a 2-year virtual fellowship program to provide mentorship and support for leadership and career development, fostering pediatric neuro-oncologists in LMICs while they remained in their home countries.

The fellowship led to the local and institutional establishment of multi-disciplinary approaches, improved patient volume, increased use of evidence-based practices, 33 scientific abstract presentations, and four article publications. The program demonstrated that pediatric oncologists in LMICs can be effectively trained in pediatric neuro-oncology, through a virtual model, enhancing the quality of care for children with CNS tumors globally. This model of training can be considered for other clinical super-specialty training to address the global challenge of specialist shortages.

## Methods

### Program Design

Designing the virtual PNO fellowship was a year-long process consisting of interviews, structure and curriculum development, and internal and external reviews. The initial stage of this process involved a series of structured interviews of 11 physicians, all of whom possessed experience providing or receiving mentorship in pediatric oncology on an international level. These physicians’ specific areas of expertise were PNO, retinoblastoma, or both. Of the 11, 6 had experience acting as mentors to trainees in LMICs; 3 had experience as mentees in LMICs; and 2 had been in both roles. All 11 had experience in pediatric oncology capacity-building in LMICs: 5 of the 11 had practiced or continue to practice in an LMIC. Interviews, 45-60 min long, consisted of a semistructured series of open-ended questions about the different aspects of global training that they provided and/or experienced and the benefits and challenges of such international distant training. Responses were mapped and recurring themes were identified.

The program’s objectives and structure were developed on the basis of interviews and analyzed feedback, leading to the creation of 3 core components of the fellowship: clinical training, mentorship, and leadership. An advisory panel for clinical training curriculum was created, consisting of an expert group of pediatric neuro-oncologists who were selected and invited based on their experience in PNO and global work. This panel reviewed the drafted curriculum and provided feedback and modified this curriculum based on their expertise.

Clinical training was designed with the objective of developing proficiency in CNS tumor management to ensure that fellows treat the full spectrum of childhood CNS tumors. Interestingly, mentorship and leadership were identified as the key components missing from most training programs. Thus, the structure of the fellowship was designed with a focus on these components.

### Clinical Training

The virtual fellowship program for clinical knowledge in CNS tumors combines structured didactics and regular mentor-mentee guidance. The initial foundation month provides foundational knowledge through readings, discussions, and prerecorded lectures.^10^^,^^11^ Didactics continued throughout the 2-year fellowship, including case discussions, tumor boards, journal clubs, and training sessions by topic experts. Fellows also attend international clinical rotations and virtual tumor boards, with opportunities for research, case logs, and networking. The program emphasizes mentor-mentee virtual interactions and requires a journal article publication for completion.

### Mentorship

Mentorship is a key component of the virtual fellowship program, so each fellow is matched with 2 mentors: a global mentor from a high-income country and a regional mentor from a local PNO program. Fellows and mentors meet regularly to set and review career and institutional goals. Bi-directional travel allows mentors to visit fellows’ sites and to understand their work environment. The program also features chief fellows who lead their cohorts, assist in planning, and collaborate with peers and program directors to enhance the educational experience.

### Leadership Development

**Figure 1.**
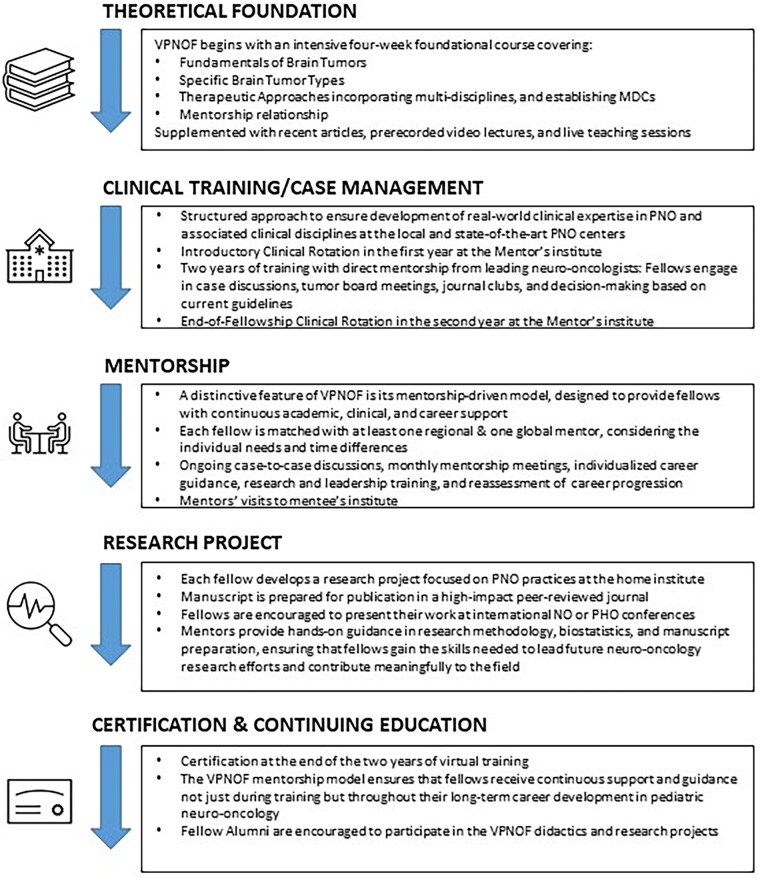
VPNOF program figure.

Effective leadership is built on strong mentorship. Without rigorous guidance, it is challenging to cultivate future leaders in the field. Therefore, significant emphasis was placed on leadership development throughout the 2-year fellowship. Both mentors of each fellow focused not only on clinical expertise but also on character development and leadership skills. This focus on leadership was further reinforced during the mentors–mentees exchange visits, providing valuable opportunities for growth and collaboration. For the consolidation of the concept of leadership, an onsite workshop at the end of the fellowship was also organized (Figure 1).

### Fellows’ Selection, Mentorship Initiation, and Didactics

Fellow selection for the program followed strict criteria, focusing on physicians with pediatric oncology training within 10 years, working at St Jude Global Alliance institutions, and showing commitment to PNO (Table 1). A scoring system was used to review applications, with final selection based on interviews. Selected fellows were matched with 2 mentors: 1 global and 1 regional, chosen from a panel of expert neuro-oncologists, selected based on thorough criteria (Table 1). Mentors provided clinical, career, and research guidance and helped with didactic training in PNO. After initial training, fellows set career and institutional goals, which were discussed and refined with mentors. Regular meetings, teaching sessions, tumor boards, and journal clubs followed, with ongoing feedback leading to the development of additional educational resources and opportunities for fellows to contribute to teaching. Fellows also maintained a case log to document diagnoses and discussions with mentors.

**Table 1.** Criteria for Fellow’s Eligibility and Application, and Mentor’s Selection Criteria

Fellow’s eligibility and application criteria	
1. Applicant criteria	
	MD or equivalent medical degree
	Fellowship—trained pediatric oncologist
	Within ten (10) years of practice post-fellowship
	Demonstrated interest in pediatric neuro-oncology
	Working in a pediatric hematology/oncology unit at a St. Jude Global Alliance member institution
2. Participating Institution Criteria	
a.	St. Jude Alliance member institution with the capacity to manage pediatric CNS tumors
b.	Human Resources
	i. Two or more pediatric oncologists
	ii. Neurosurgeon with training and/or experience in pediatrics
	iii. Pathologist with experience in neuropathology
	iv. Radiologist with training and/or experience in pediatric neuroradiology
c.	Diagnostic and Treatment Resources (or local accessibility if not at institution)
	i. Basic Chemotherapy
	ii. Computed Tomography (CT)
	iii. Magnetic Resonance Imaging (MRI)
	iv. Radiation therapy
	v. Pediatric Intensive Care Unit (ICU)
3. Required Documents	
a.	Brief personal statement describing personal and professional goals as they relate to training in pediatric neuro-oncology, and goals for building capacity for pediatric neuro-oncology service at the applicant’s hospital
b.	Curriculum Vitae
c.	Medical school diploma
d.	Documentation of pediatric oncology residency completion
e.	Letters from:
	i. Department head indicating need within the department for a pediatric neuro-oncology trained physician and support for the applicant’s participation in the fellowship program, including time commitment.
	ii. Letter of recommendation from a supervising physician familiar with your work, separate from the department head

Mentor's selection criteria	
1. A renowned Pediatric Neuro-Oncologist working at a specialized center	
Global Mentor	Preferably from an HIC, with experience in global health work
Regional Mentor	Either from an HIC or an LMIC, with a background of regional understanding of the social and healthcare context, with the mentee
2. Can dedicate an average of 1-2 hours/week time commitment (aside from clinical rotations)	
i.	Participate in a two-hour virtual workshop for mentorship preparation
ii.	Facilitate a virtual tumor board every 2-3 months
iii.	Mentee advising (clinical and research project guidance, and career mentorship)
	Via email, calls, and messages on an as-needed basis, less frequent over time
	Monthly mentorship meetings
iv.	Intermittent evaluation: Feedback emails/calls with the St. Jude Global Neuro-Oncology team
	Monthly for the first 3 months, then quarterly for the remainder of the period
v.	Provide occasional didactics:
	Cases for discussion via email or for a case bank
	Occasional teaching sessions (once per year)
3. Mentee’s Clinical Rotations and Site Visits	
i.	May host a trainee at their own institution
	4 weeks during the first year (Preferably at the Regional Mentor’s site)
	4 weeks during the second year (Preferably at the Global Mentor’s site)
ii.	Visit to the trainee’s institution
	A 3–5 day visit to the trainee’s institution, duration based on their schedule
	Submission of the visit summary upon return

### Conferences and Workshops

The St Jude VPNOF supports annual travel for fellows to regional and international conferences, requiring them to submit abstracts based on retrospective reviews with mentor guidance. Fellows are encouraged to attend conferences such as SIOP and ISPNO, as well as regional events that benefit their institutions. St Jude also funds team members’ travel for conferences focused on team building and supports fellows’ attendance at specialized courses and workshops, such as the PBII Pediatric Neuro-Oncologic Imaging Course, Neuro-Oncology Training Seminar, and Leadership Workshop.

## Results

**Figure 2.**
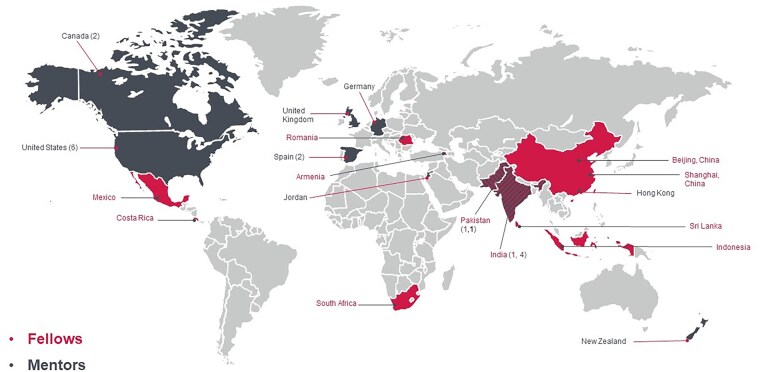
Fellows-Mentors world map.

Eleven fellows have been selected in the first 2 cohorts: 5 began the 2-year program in September 2022; and 6, in September 2023. These eleven fellows represent 11 cities in 10 countries across the world. Each of the fellows was matched to 1 global and 1 or 2 regional mentors, with a total of 20 mentors (Figure 2).

Assessment of baseline institution resources of each of the inducted fellows was done at the beginning of the fellowship (Table 2). After 24 months of the first cohort of fellows and 12 months of the second cohort, the fellows had each managed, discussed with mentors, and logged an average of 35.9 patients’ cases [Range, 14-80; Median, 34] (Table 3).

**Table 2.** Baseline assessment of institution resources of fellows

	2022					2023					
Name of the country	Armenia	China (Shanghai)	Indonesia	Mexico	Pakistan	China (Beijing)	Costa Rica	India	Romania	South Africa	Sri Lanka
**No. of pediatric oncologists**	8	10	7	7	6	15	7	4	10	5	3
**No. of pediatric neuro-oncologists (formally trained)**	1	3	0	1	0	0	1	0	0	1	0
**No. of trained pediatric neurosurgeons/or with experience**	5	8	3	2	1	15	3	2	4	3	1
**No. of pathologists with experience in neuropathology**	2	2	1	1	1	3	1	3	1	1	2
**SNo. of trained pediatric neuro-radiologists/or with experience**	2	2	1	1	2	4	0	2	1	1	0
**No. of radiation oncologists**	1	3	4	1	2	0	3	2	5	1	15
**No. of endocrinologists**	2	4	2	3	2	10	3	1	2	4	2
**No. of ophthalmologists**	1	3	6	5	2	10	5	9	2	10	3
**No. of clinical pharmacists**	0	3	4	1	1	18	3	0	0	0	0
**Patient/nurse Ratio**	6:1	7:1	5:1	3:1	12:1	2:3	8:1	4:1	10:1	6:1	8:1
**No. of PICU beds for neurosurgery patients**	1	1	3	2	8	5	3	2	2	2	4
**Nurse coordinator**	No	Yes	No	Yes	No	Yes	Yes	No	No	No	No
**Social worker**	Yes	Yes	No	Yes	Yes	Yes	Yes	Yes	Yes	Yes	No
**Palliative care specialist/services**	Yes	Yes	Yes	Yes	Yes	Yes	Yes	Yes	No	Yes	Yes
**Psychologist**	Yes	No	No	Yes	Yes	Yes	Yes	Yes	No	Yes	Yes
**Audiologist**	No	Yes	Yes	Yes	Yes	Yes	Yes	Yes	Yes	Yes	Yes
**Physical therapist**	Yes	Yes	Yes	Yes	Yes	Yes	Yes	Yes	No	Yes	Yes
**Occupational therapist**	No	No	Yes	Yes	Yes	No	Yes	Yes	No	Yes	Yes
**Existing institutional neuro-Oncology MDT**	Yes	Yes	Yes	Yes	Yes	No	Yes	Yes	No	Yes	No
**Existing institutional referral system between the neurosurgeons & oncologists**	Yes	Yes	No	Yes	No	No	Yes	Yes	No	Yes	Yes
**Standard of care treatment protocols for brain tumors at the hospital**	Yes	Yes	No	Yes	No	No	Yes	No	No	No	Yes

**Table 3.** Total number of cases logged from September 2022 to September 2024

TYPE of pediatric CNS tumor	*N*
Low-grade glioma	129
High-grade glioma	62
Medulloblastoma	58
Other embryonal tumors	14
Ependymoma	40
Germ cell tumors	28
CPP/CPC	11
Craniopharyngioma	13
Pineoblastoma	9
Other CNS tumors	31
TOTAL	**395**

Abbreviations. CNS: *central nervous system*; CPP: *choroid plexus papilloma*; CPC: *choroid plexus carcinoma*.

The clinical rotations to the mentor site were carefully designed by the fellows, their mentors, and the program directors to meet their personal and institutional needs. During the continuous development of the program, the timing and duration of the clinical rotations were made flexible for maximum benefit. From September 2022 until September 2024, 14 clinical rotations occurred. The median duration of each clinical rotation was 26.5 days (Range, 14-34 days).

At the data cut-off point, seven 2- to 6-day regional and global mentor visits to the mentee sites had occurred. They advocated meetings with the hospital and departmental leadership and multi-disciplinary talks to further their collaboration. According to the fellows’ feedback, the mentor visit was the most impactful component of the fellowship in terms of a better understanding by the mentor of their work, cultural environment, and resources, serving to improve multi-disciplinary team cooperation and increase team trust after the visit.

As a result of conference support and attendance, fellows have published four peer-reviewed manuscripts, with ­several others in progress. Additionally, 33 abstracts have been accepted at conferences, including 3 for oral presentations.

**Figure 3.**
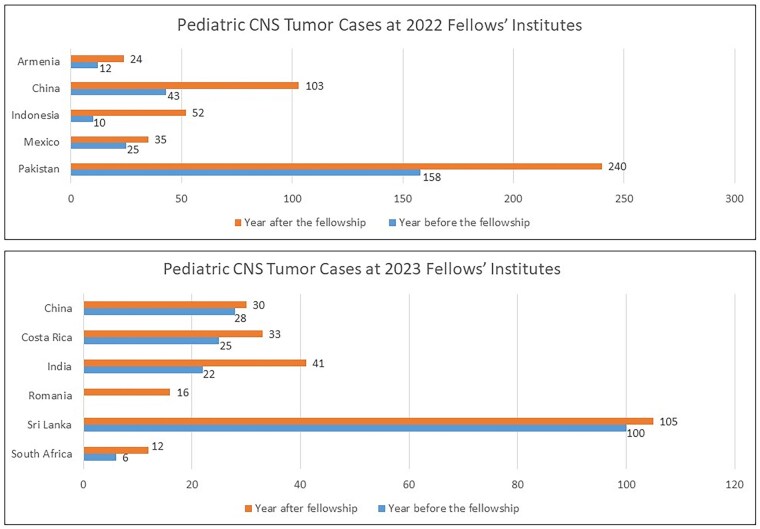
Bar graphs 2022 2023.

The majority of fellows reported an increase in the referrals of PNO patients to the respective institutes after the enrollment in the fellowship program, which was evident in the registry of cases (Figure 3). Fellows consistently worked with the fellowship directors and their global and regional mentors toward their career and institutional goals, with the ultimate objective of building CNS tumor management capacity. Multidisciplinary care team building and strengthening was consistently seen as a goal among all fellows at different levels, and this goal was supported through consistent virtual sessions with pediatric neuro-oncologists, neurosurgeons, radiation oncologists, and radiologists. Six of the 11 fellows reported that they developed the first multidisciplinary PNO team at their respective hospitals, including initiating multidisciplinary care team tumor boards. Moreover, one fellow reported the development of a national-level tumor board. The remaining 5 fellows noted the enhancement of the existing teams with subspecialists.

Five fellows reported establishing a hospital-based CNS tumor registry. One fellow established the first national PNO registry, and one was able to centralize the neurosurgery services in their city. One initiated the establishment of a methylation facility at her institute with the assistance of her mentor. One, with the support of St Jude Global, organized the first hybrid PNO symposium in the province, attracting approximately 100 onsite and online participants. Two fellows started sending tissue samples from their institutes to the mentors’ sites for molecular testing.

Although all the fellows mentioned that the program structure is ideal, they still experienced a few challenges. The major challenge areas were inadequate knowledge in associated subjects such as neuroradiology, anatomy, and radiation therapy (10 fellows); lack of protected time for virtual fellowship (8 fellows); clinical burden of hematology and other solid tumors (7 fellows); lack of support from the local oncology team (5 fellows); and difficulties collaborating with the local multidisciplinary team (4 fellows). They also mentioned language barriers (4 fellows), visa issues (3 fellows), traveling restrictions (3 fellows), time zone differences (2 fellows), clinical rotations (2 fellows), internet limitations (2 fellows), and family issues (2 fellows).

## Discussion

Sub-specialty training in LMICs faces numerous inherent challenges, including the scarcity of local fellowship opportunities, limited scholarships for training abroad, brain drain, and the tendency of trained specialists not to return to their home countries after completing their education in higher-income nations. Furthermore, there is a lack of regional sub-specialized societies in LMICs, as well as limited institutional and international support for the few sub-specialists working in these regions.

Our core philosophy in this fellowship program is to succeed in academia, institutional support, and international mentorship. Consequently, the primary aims of St Jude Global Neuro-Oncology are to identify and collaborate with motivated professionals in LMICs, secure institutional backing, and facilitate or fund international mentorship and support.^12^

The fellows of the VPNO program often describe the experience as “a dream come true.” One of the most significant aspects of the fellowship is the opportunity to establish direct, one-on-one connections with global leaders in PNO. As one fellow aptly stated, “We gained access to the celebrities in PNO.” The chance to ask questions at any time and from any location has proven to be an invaluable experience. The fellows consistently highlighted their exposure to international, innovative treatment practices, which not only enhanced their knowledge but also increased their confidence in managing PNO patients. Additionally, they were able to foster strong professional relationships with regional and global mentors, as well as peers across various institutions, countries, and regions from the start of the program. The fellows also expressed that the program exceeded their expectations. They greatly valued the research mentorship provided by their global and regional mentors, as well as the guidance from the VPNOF program directors. The opportunity to present their research to a global audience was described as a particularly impactful experience.

The fellows also developed essential team-building and leadership skills. Every fellow in the first cohort reported that joining the virtual fellowship enhanced their recognition as trained professionals in the field. Since the initiation of the virtual training, substantial improvements have been made in local and institutional PNO services. All fellows acknowledged that their virtual fellowship experience directly contributed to advancements in the care of PNO patients within their institutions. The use of peer review and a multidisciplinary management approach led to a significant increase in the registration of pediatric CNS tumor cases in the fellows’ settings, resulting in the provision of optimal care for these patients. These important changes are expected to improve survival rates and reduce disease-related and treatment-associated morbidity and mortality in LMIC settings. Additionally, the fellows’ improved registry services and data management will support future quality improvement initiatives. The fellows were provided with a valuable opportunity to attend conferences and showcase their research. The papers published by the fellows from LMICs have contributed valuable perspectives from resource-limited environments to the global scientific literature.

The training program faced several limitations such as the significant time zone differences posed challenges for real-time communication and coordination between international participants. Additionally, securing approval for 2extended travel periods for clinical rotations, each lasting a month, was a hurdle for some fellows, as obtaining permission from their supervisors proved difficult. Organizing exchange visits between mentors and mentees was also challenging due to the need to align schedules across different time zones and professional commitments. Language proficiency issues further hindered communication and understanding for some fellows, adding another layer of complexity to the training. Resolving conflicts remotely proved to be another obstacle, as it was harder to address issues effectively without in-person interaction. Furthermore, navigating local institutional politics and regulations presented an additional barrier, requiring fellows to adapt to differing organizational norms. Lastly, visa-related issues, such as securing the necessary travel documents to attend conferences and on-site teaching sessions, delayed or prevented some fellows from participating.

Most of the challenges and limitations were addressed, as mentioned in the results section, but as the program evolved, many of these limitations were resolved with extra resources based on an individual tailored approach according to fellows’ needs and mentors’ feedback. The fellows’ knowledge gaps were addressed through additional focused teaching in areas such as neuroradiology, radiation oncology, palliative care, and neurosurgery. Clinical rotations were scheduled based on the availability and convenience of both the fellows and their mentors. The program directors had regular conversations with the fellows, their mentors, and the institution’s leadership about time management and any local challenges. Additionally, the co-director and chief fellows held frequent group and one-on-one meetings with the fellows to discuss both academic and personal matters, ensuring that all concerns were addressed in the best possible way. Over time, with continued support from the St Jude Global Neuro-Oncology team and the growth of neuro-oncology services at the fellows’ institutions, the fellows were given more protected time for neuro-oncology work. Language barriers were addressed through customized language training. The evaluation of this program’s impact on improving overall survival, morbidity, and mortality rates of pediatric CNS tumors in LMICs was limited due to the short study duration. However, based on the experience of leading centers in LMICs, such as King Hussein Cancer Center in Jordan, we can confidently say that these changes will directly enhance the care, prognosis, and quality of life for children with CNS tumors. Our ongoing virtual training experience shows that it is an effective way to prepare specialists in LMICs and strengthen local healthcare capacity. This training model could also be applied to other subspecialties to help address other global health challenges.

## Supplementary Material

vdaf229_Supplementary_Data

## Data Availability

All the data collected for the study are included in the results, and supplementary material will be made available with publication, including the de-identified participants’ data.
