# Supplementary material for: An innovative virtual fellowship leveraging global and regional mentorship to foster pediatric neuro-oncologists in low/middle-income countries
Source: Neurooncol Adv. 2025 Oct 23;7(1):vdaf229. doi: 10.1093/noajnl/vdaf229 (PMC12746595; doi:10.1093/noajnl/vdaf229)
Supplement: vdaf229_Supplementary_Data [file vdaf229_supplementary_data.docx]

**SUPPLEMENTARY APPENDIX**

*METHODS*

***Clinical training***

Virtual teaching was designed to improve clinical knowledge in CNS tumors through both structured group didactics and regular mentor-mentee clinical advice on all new, relapsed, and complicated cases treated by the virtual fellows. An initial month of didactics, known as the Foundation Month, was created to ensure that all trainees began the program with a baseline of foundational knowledge of the most common pediatric CNS tumors as well as multi-disciplinary care of those with CNS tumors (Figure 4). Over the first three weeks of the Foundation Month, reading assignments of peer-reviewed literature and textbook chapters were given, and each week a facilitated discussion about specific topics occurred. Textbooks provided by St. Jude Children’s Research Hospital were shipped to the fellows by the VPNOF program immediately after fellows were selected. All other reading materials and pre-recorded video lectures were organized and made available in a virtual classroom on the St. Jude Cure4Kids platform. [1, 2]

Following the Foundation Month, virtual didactics continued throughout the two-year fellowship, generally twice per month. Didactics consisted of various styles according to topic and learning objective. The most frequent didactics involved case discussions and occurred twice a month. Furthermore, training on rare tumors or interesting cases was sent to the fellows by email. A clinical case of a child with a CNS tumor that was being managed by one of the fellows was presented in a 60-minute session in a “tumor board” style on a St. Jude Global tumor board template, followed by discussion and teaching facilitated by one or both of the fellow’s mentors and the program directors. These presentations occur on a rotating basis so that each fellow presents several times throughout their training.

Monthly or twice-monthly teaching sessions based on a preset list of neuro-oncology and related topics are presented by topic experts. A journal club is presented six times per year —once by each second-year fellow— and these are prepared in advance under mentor guidance. Fellows are also required to attend the St. Jude Global Academy Pediatric Neuro-Oncology (GAPNO) international virtual tumor board, which is one of the main St. Jude virtual training initiatives for PNO [3], and to select at least one more international tumor board provided by other major PNO programs to attend regularly. Fellows are required to attend a minimum of 80% of all virtual didactics, allowing some flexibility given time zones and institutional responsibilities.

Moreover, fellows were provided with in-person international training opportunities during the program: They were required to attend two clinical rotations (one every year of the fellowship) at their regional mentor’s site. Fellows traveled to the regional mentor’s site for two four-week rotations to meet with them and to observe the clinical service and experiences in related disciplines. This was designed with different goals for each rotation: for the initial rotation, scheduled within the first three months of the fellowship program, the primary goals are a focus on fundamental clinical knowledge acquisition, as well as meeting their mentor and establishing the mentor relationship. The second rotation, scheduled to be within the last six months of the two-year program, aims to provide a “consolidation of knowledge and leadership skills” as well as an opportunity to observe and learn systems and capacity building. The cost of the visit is funded by the St. Jude Global Neuro-Oncology program.

Further, clinical teaching was provided by both mentors to the fellow via mentor-mentee virtual meetings on an ad hoc, as-needed basis on predetermined platforms such as phone, message, Zoom, and email, enabling the mentee to seek clinical advice on real-time while managing patients in their home institution. The fellows documented these cases and discussion points in a case log, which must meet pre-set requirements of a minimum number and type of CNS tumor diagnoses, and completion of at least 80% of these by the end of two years. (Table 4)

Additionally, other in-person experiences take place through research, as fellows are required to perform a retrospective review of aspects of PNO care at their institution for submission to an international conference at which they meet their mentor(s) in-person again. Mentors and program directors provided research and writing guidance, and fellows were sponsored to attend the conference, which also provided networking opportunities to meet with their mentors, other international PNOs, and their VPNOF cohort. After feedback from the first cohort of fellows, one paper publication in an indexed medical journal was added as a requirement for successful completion of the fellowship.

***Mentorship***

Mentorship is the major component of the virtual fellowship program. Upon acceptance, each fellow was matched to two mentors to provide career and clinical guidance during the two-year program and beyond. Each fellow was matched to one global mentor, typically from a high-income country, and one regional mentor from a local PNO program with similar context whenever possible. A session was facilitated to get to know one another, to understand the clinical setting that the trainee works in, and to set career goals for the trainee and capacity-building goals for their institution.

Mentors and mentees then met at a pre-set frequency (minimum monthly, typically more frequently) to discuss progress toward the goals previously set; these meetings were separate from the *ad hoc* clinical advice meetings. In addition to clinical and mentorship meetings, we built in bi-directional travel opportunities for mentors and fellows. Moreover, both regional and global mentors were supported in visiting the fellow’s site to better understand their work environment and culture, to serve as an advocate for them on their path toward building their own program and to encourage/facilitate leadership consolidation. The mentors’ visits were sponsored by the St. Jude Global Neuro-Oncology program. Because mentorship was identified early as a main prerequisite for success, the St. Jude Global Neuro-Oncology team added another layer of mentorship. Additionally, the system of two chief fellows in the second year of fellowship from each cohort was also launched, and the chief fellows (JH and RA) from the 2022 cohort were selected. The current chief fellows (TN and MS) are from the 2023 cohort. These fellows were selected as head of fellows of the program and collaborated in preparing the monthly agenda, one-on-one meetings with their peers, advising on case presentations, and helping to resolve challenges arising in the program. Chief fellows had direct communication and biweekly meetings with the program directors to improve and optimize the educational process in a comprehensive manner.

***Fellow and Mentor Selection***

Fellow selection was based on strict inclusion criteria: physician with formal training in pediatric oncology; within ten years of completion of such training; at an institution with membership in the St. Jude Global Alliance (<https://global.stjude.org/en-us/global-alliance/about.html>); and has committed institutional support in PNO. A selection committee was created that reviewed the *applications and interviewed candidates*. Fellows’ selection was based on existing collaboration with St. Jude Neuro-Oncology , existing basis disciplines for NO care (Rad, path, RO), membership alliance, track record of basic NO services, patient volume to build up MDT, regional needs consideration, personal…? Final selection was based on the interview with the mentors and supervisors.

Once trainees were selected, a process of matching each trainee to two mentors occurred. Mentors were selected from an expert panel on the basis of the following inclusion criteria: well-known neuro-oncologist, recommendation to the panel by a colleague, working in or training someone in an LMIC, didactic experience, and good research track. An initial pool of 25 mentors was created; all committed to volunteering their time and expertise to the long-term mentorship and training of a pediatric oncologist in a low-resource country. All mentors were requested to complete a survey to better understand their mentorship experience, specific areas of expertise, geographic experience, and availability. Mentors served three roles: to provide clinical and career mentorship to their selected fellow; to provide didactics to the trainee groups in their specific areas of interest and expertise within neuro-oncology; and to recruit clinicians in related disciplines to assist in teaching and clinical advice (e.g. radiation oncology, neurosurgery). Fellows were matched to two mentors from the expert panel: a “global mentor” and a “regional mentor.” The global mentor was typically in a high-resource country but with experience or connection to practice in LMICs. The regional mentor was selected from the same geographic region as the trainee so the culture and environment of the trainee would be understood, enabling provision of contextually relevant guidance.

***Mentorship initiation and didactics***

During the fourth and final week of the Foundation Month, fellows and mentors attended a joint mentorship workshop. Prior to the session, literature on mentorship practices was reviewed by all attendees. This advanced preparation allowed thoughtful discussion of setting expectations and responsibilities of both mentors and mentees.

Fellows also completed a worksheet in advance, describing their background, experience, needs, challenges, and goals; breakout sessions of each mentorship triad (fellow, regional mentor, and global mentor) enabled review of this form to better understand the fellow’s environment and context. This led to shared goal setting: each fellow presented two or three goals for their personal career as a PNO and two or three goals for capacity-building in their institution. These goals were discussed with the mentors and co-designed, then presented for discussion amongst all fellows and mentors for further refinement. This session marked the beginning of the formal mentorship relationship. Following the establishment of this relationship, regular monthly meetings were set to review and discuss progress toward the goals.

Following the Foundations Month, an evaluation survey was distributed, and recurring feedback demonstrated that the fellows requested additional didactics in the form of teaching sessions. A topics list was developed collaboratively between fellows and program directors, ensuring that it covered standard PNO topics while reflecting topics that the fellows requested to learn more about, such as immunotherapy and strengthening their radiology skills. Experts in all topics were recruited to teach in a mixed format of lecture and question-and-answer, ensuring an opportunity for fellows to interact and ask questions specific to their needs. Twenty-four expert and 6 fellow teaching sessions occurred before September 2024. In recent months, these have evolved to allow fellows to occasionally teach with the support of their mentor. Biweekly tumor boards were also conducted. From September 2022 until September 2024, thirty-eight cases have been presented and discussed through this approach. In response to feedback, journal club sessions have been included intermittently since November of 2023 to introduce an additional element of critical analysis of medical literature. As of September 2024, five fellows had presented at the journal club after guidance by their mentors.

The case log, a file provided by St. Jude Global, requires the fellow to log diagnosis, initial management steps, and key points discussed with mentors. The mentors then sign off on all cases discussed.

***Conferences and workshops***

The St. Jude VPNOF supports travel on an annual basis to various regional and international conferences and workshops. Fellows are required to prepare and submit an abstract to a conference based on a retrospective review performed with the guidance of their mentors and the program directors. Additionally, the VPNOF provides specific international conference opportunities (International Society of Pediatric Oncology [SIOP] and International Society of Pediatric Neuro-Oncology [ISPNO]) and supports fellow attendance at loco regional conferences that may be impactful for their institution. Moreover, St. Jude supports travel of multi-disciplinary team members to regional conferences when team building is one of the main fellow goals. St. Jude also supported the fellows’ attendance at “Pediatric Biomedical Imaging Initiative (PBII) Pediatric Neuro-Oncologic Imaging Course” in October 2022 and the Neuro-Oncology Training Seminar in October 2022 and April 2024, and a leadership workshop in the end of training. At these conferences, fellows present their research; meet with one another, their mentors, and the program leadership to network; and discuss their progress and challenges individually as well as in a group setting.

Figure 4. A flowchart summarizing the two-year journey

**
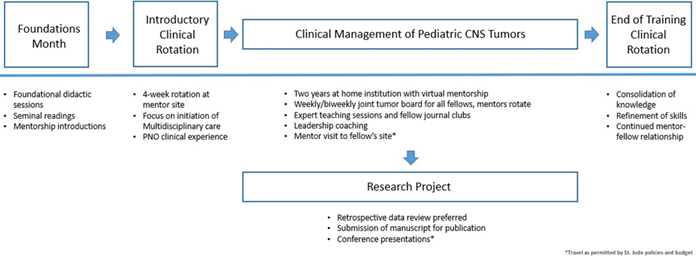
**

Table 4. Pre-set requirements of a minimum number and type of CNS tumor diagnoses

| **DIAGNOSIS** | **# CASES** |
| --- | --- |
| Low-grade glioma outside the optic pathway, including 1 metastatic | 2 |
| Optic pathway glioma | 2 |
| Diffuse midline glioma | 2 |
| High-grade gliomas outside the brainstem | 2 |
| Ependymoma: 1 posterior fossa, 1 supra tentorial | 2 |
| Medulloblastoma: 1 standard or high-risk, 1 infant group | 2 |
| Other embryonal tumors: ATRT, Pineoblastoma, sPNET | 3 |
| Germ cell tumor: 1 germinomatous, 1 non-germinomatous | 2 |
| Craniopharyngioma | 1 |
| Infant brain tumors, any pathology, including EPN and MB | 3 |
| Primary spinal tumor | 1 |
| Relapsed brain tumor, NOS | 2 |
| **TOTAL**  **80% of total^*^** | **24**  **19** |

Abbreviations. ATRT: Atypical Teratoid/Rhabdoid Tumor; sPNET: Supratentorial Primitive Neuroectodermal Tumor; EPN: Ependymoma; MB: Medulloblastoma; NOS: Not otherwise specified.

*Minimal mandatory number of cases for the fellowship

**References:**

1. Berg F, Conger K, Avula M, et al. The transformation of Cure4Kids: Expanding knowledge transfer capacity. Pediatr Blood Cancer. 2024;71(4):e31135. doi:10.1002/pbc.31135
2. Moreira DC, Jones HM, Schaeffer E, et al. Cure4Kids: Two decades of knowledge transfer. Pediatr Blood Cancer. 2022;69(3):e30007. doi:10.1002/pbc.30007
3. Moreira DC, Gajjar A, Patay Z, et al. Creation of a successful multidisciplinary course in pediatric neuro-oncology with a systematic approach to curriculum development. Cancer. 2021;127(7):1126-1133. doi:10.1002/cncr.33310
